# Sport-related concussions

**DOI:** 10.1590/S1980-57642014DN81000003

**Published:** 2014

**Authors:** Jéssica Natuline Ianof, Fabio Rios Freire, Vanessa Tomé Gonçalves Calado, Juliana Rhein Lacerda, Fernanda Coelho, Silvia Veitzman, Magali Taino Schmidt, Sergio Machado, Bruna Velasques, Pedro Ribeiro, Luis Fernando Hindi Basile, Wellingson Silva Paiva, Robson Amorim, Renato Anghinah

**Affiliations:** 1 Center of Cognitive Rehabilitation after Traumatic Brain Injury of the Neurology Department of Hospital das Clinicas of the University of São Paulo, São Paulo, Brazil.; 2 Neurology and Neurosurgery Service at Hospital Samaritano, São Paulo, Brazil.; 3 University Salgado de Oliveira, Niterói - RJ and Panic and Respiration Laboratory, (IPUB/UFRJ), Rio de Janeiro, Brazil.; 4 Laboratory of Brain Mapping and Sensory-Motor Integration (IPUB/UFRJ), Rio de Janeiro, Brazil.; 5 Laboratory of Psychophysiology, School of Health, University Metodista of São Paulo, São Paulo, Brazil.; 6 Division of Neurosurgery, University of São Paulo Medical School, São Paulo, Brazil.

**Keywords:** TBI, traumatic brain injury, concussion, sports

## Abstract

Traumatic brain injury (TBI) is a major cause of lifelong disability and death worldwide. Sport-related traumatic brain injury is an important public health concern. The purpose of this review was to highlight the importance of sport-related concussions. Concussion refers to a transient alteration in consciousness induced by external biomechanical forces transmitted directly or indirectly to the brain. It is a common, although most likely underreported, condition. Contact sports such as American football, rugby, soccer, boxing, basketball and hockey are associated with a relatively high prevalence of concussion. Various factors may be associated with a greater risk of sport-related concussion, such as age, sex, sport played, level of sport played and equipment used. Physical complaints (headache, fatigue, dizziness), behavioral changes (depression, anxiety, irritability) and cognitive impairment are very common after a concussion. The risk of premature return to activities includes the prolongation of post-concussive symptoms and increased risk of concussion recurrence.

## Abstract (pt)

O traumatismo cranioencefálico (TCE) é uma das principais causas de deficiência ao longo da vida e de morte no mundo. As lesões cerebrais traumáticas relacionadas ao esporte são um problema de saúde pública de grande impacto. O intuito desta revisão foi expor a importância das concussões relacionadas ao esporte. A concussão é uma alteração transitória da consciência induzida por forças biomecânicas externas que podem ser direta ou indiretamente transmitidas ao cérebro. Sua ocorrência é comum, mas provavelmente é subnotificada. Esportes de contato, como futebol americano, rugby, futebol, boxe, basquetebol e hóquei, estão associados a uma prevalência relativamente alta de concussão. Diferentes fatores podem estar associados a um maior risco de concussão relacionada ao esporte, como idade, sexo, esporte jogado, categoria de esporte jogado e equipamentos utilizados. Queixas físicas (dores de cabeça, fadiga, tonturas), alterações comportamentais (depressão, ansiedade, irritabilidade) e comprometimento cognitivo são muito comuns após uma concussão. O risco de retorno prematuro às atividades inclui o prolongamento dos sintomas pós-concussionais e o aumento do risco de recorrência de concussão.

## INTRODUCTION

Traumatic brain injury (TBI) is an insult to the brain from an external mechanical force, which can lead to permanent or temporary impairment of cognitive, physical, and psychosocial functions.^1^

The major causes of TBI include motor vehicle accidents (50%), falls (21%), assaults and robberies (12%), and accidents during practice of sports/leisure activities (10%).^2^

Sport-related traumatic brain injury is an important public health concern and is often labelled as a 'silent epidemic'. Estimates suggest that 1.6-3.^8^ million sport-related TBIs occur annually in the USA, and this number includes injuries for which no medical care is sought.^3^ However, many sport-related TBIs are unrecognized and unreported. Sports that involve contact and/or collisions, such as boxing, American football, ice hockey, soccer, rugby and martial arts, as well as high-velocity sports such as cycling, motor racing, equestrian sports, rodeo, skiing and roller skating, are associated with an increased risk of TBI.^4^

TBI can generally be classified as acute or chronic. Acute TBI is used to describe injuries that occur immediately at the time of impact, with subsequent signs and symptoms of TBI, whereas chronic TBI refers to the long-term consequences of single or multiple brain traumas.^4^ In this review, we will focus on concussion, a very common acute brain injury.

The aim of this review was to highlight the importance of sport-related concussions.

**Acute traumatic brain injury - brief background.** There are a variety of acute TBI pathologies that may occur in athletes involved in high-risk sports. The most common acute brain injury in athletes is cerebral concussion. Focal brain injuries, diffuse axonal injury, skull fractures and penetrating brain injury - moderate and severe injuries - are less common in sports.^5^

**Concussion.** Concussion is a complex pathophysiological process that affects the brain, induced by traumatic biomechanical forces.^6^

A concussion occurs following transmission of direct or indirect impulsive forces to the head, resulting in short-lived neurological impairments.^6-8^ Cognitive, physical and behavioural signs and symptoms manifest. Memory impairment, headache and dizziness are very common symptoms. Loss of consciousness is not a requirement for diagnosis of concussion. Most concussions in adults tend to resolve spontaneously (within 7-10 days);^9-13^ although the recovery period can be longer in children and young adolescents.^8^

Conventional structural neuroimaging may not be able to detect structural injury but the clinical symptomatology reflects a functional disturbance.^6-8^

**Mechanisms of injury.** Biomechanical forces that are capable of causing brain injury are probably a combination of rotational, linear and/or impact decelerations. Impact deceleration occurs when the head rapidly decelerates, typically when the head strikes a playing mat or field, or an arena floor. It can also occur when an athlete's head rapidly decelerates upon striking the body of an opposing player or fixed structures such as a goalpost, railing, tree or hockey board.^4^

Closed head injury with acceleration and deceleration forces to the brain causes a multifaceted cascade of neurochemical changes that affect brain function. Although detailed understanding of the pathophysiology of concussion is lacking, studies using the mild fluid percussion model support the idea that the initiating event involves the stretching and disrupting of neuronal and axonal cell membranes, leaving cell bodies and myelin sheaths less affected.^14^ These processes lead to membrane defects, causing a deregulated flux of ions, including an efflux of potassium and influx of calcium. These events precipitate enhanced release of excitatory neurotransmitters, notably glutamate. Binding of glutamate to N-methyl-D-aspartate (NMDA) receptors results in further depolarization, influx of calcium ions, and widespread suppression of neurons with glucose hypometabolism.^15,16^

Increased activity in membrane pumps - in order to restore ionic balance - raises glucose consumption, depletes energy stores, causes calcium influx into mitochondria, and impairs oxidative metabolism and consequently anaerobic glycolysis with lactate production.^15,16^

Additional cascades or processes may then initiate or result, such as apoptosis, calpain-caspase activation, mitochondrial dysfunction, free radical formation, neuroinflammation, growth factor alterations, inflammatory processes^17^ and amyloid cascade.^18^

## DISCUSSION

**Factors affecting concussion risk for athletes.** A number of factors may lead to greater risk of sport-related concussion. There is insufficient evidence to affirm that age or level of competition affects (increasing or decreasing) the risk of concussion.^19-22^

Since there are more male participants in sports, the absolute number of concussions is higher in men. However, the relationship of concussion risk and sex varies across sports. The sports with the highest risk for men are football and hockey;^23-26^ and for women are soccer and basketball.^26,27^

With the exception of combat sports (like boxing and mixed martial arts - MMA), American football and Australian rugby most likely pose a greater risk of concussion than other sports.^19,23,24^ The risk is probably lowest in baseball, softball, volleyball, and gymnastics.

Regarding equipment, it is highly likely that headgear use has a protective effect on concussion incidence in rugby.^28,29^ Similarly, mouth guards do not seem to protect athletes from concussion.^28,30^ There is a lack of evidence to support or refute the efficacy of protective soccer headgear. Also, data are insufficient to support or refute the superiority of one type of football helmet over another in preventing concussions.

There is insufficient data to characterize concussion risk by position in most major team sports. Linebackers, offensive linemen, and defensive backs probably have greater risk of concussion than receivers in college football.^30,31^ The risk of concussion is increased by body checking in ice hockey.^32^

Factors related to the athlete, such as body mass greater than 27 kg/m^2^ and training time of less than 3 hours weekly seem to increase the risk of concussion.^33^

**Detection and diagnosis.** Currently, there is no device that enables clinical diagnosis of concussion.

Neuropsychological testing can assist toward determining the occurrence and resolution of cognitive impairment.^4^

Usually, the standard structural neuroimaging outcomes are typically normal in patients who are evaluated for sport-related concussions.^6-8^ However, new structural, functional and/or metabolic imaging technologies may be useful for detection of subtle structural or functional brain injury.

There is controversy over the usefulness of diffusion tensor imaging (DTI) in the evaluation of acute concussion.

Luther et al. (2012) observed decreased fractional anisotropy (suggesting reduced fibre-tract integrity) in one out of 11 tracts in professional American football players with concussion. However, no abnormalities on susceptibility-weighted imaging (SWI) - indicative of prior microhemorrhages - were found.^34^ By contrast, a case study of a concussed athlete reported significant and co-localized changes in fractional anisotropy and mean diffusivity (suggestive of axonal injury) voxels in the right corona radiata and right inferior longitudinal fasciculus.^35^

Athletes with concussion have altered activation patterns revealed by functional MRI (fMRI), compared with controls. Concussed players exhibited increases in the amplitude and extent of blood oxygen level-dependent activity, indicating high levels of brain activity. In addition, athletes who exhibited hyperactivation on fMRI had a more prolonged clinical recovery.^36^

A noninvasive technique that can be used to identify neurometabolic changes in the acute post-concussion phase is magnetic resonance spectroscopy. Decreased levels of glutamate in the primary motor cortex and decreased levels of *N*acetylaspartate (marker of neuronal integrity) in the prefrontal and M1 cortices were found in concussed athletes.^37^

**Figure 1:**
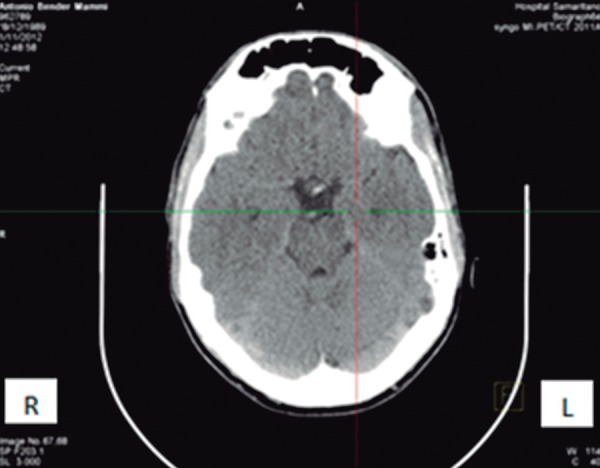
Cranial CT scan. No abnormalities were found.

**Figure 2:**
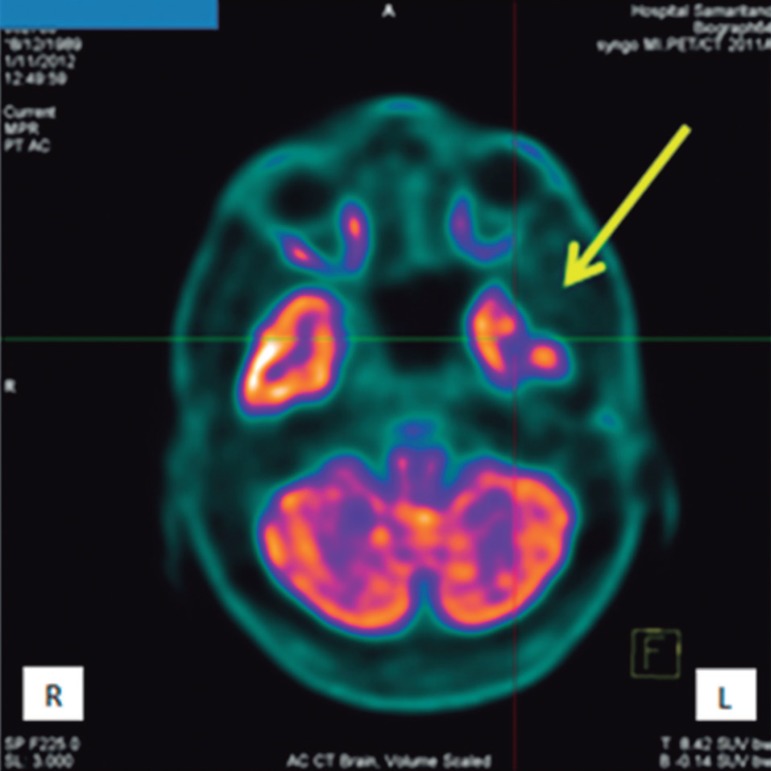
Brain PET/CT. The arrows indicates decreased metabolism of glucose in the left temporal region.

**A case to illustrate the diagnosis.** A 23-year-old man, who suffered a concussion that did not involve loss of consciousness, was attended at our service in 2013. While playing soccer, he was struck on the left temporal region of his head by another player's knee. He had time disorientation for a few minutes and was also suffering with a posttraumatic headache. He sought medical help at our Emergency Service four hours later, and described a mild-moderate headache. The patient was submitted to a magnetic resonance imaging of the brain (MRI) and cranial computed tomography (CT) scan (Figure 1). No abnormalities were found on either of the exams. After these results, he was submitted to a brain positron emission tomography-computed tomography (PET/CT), which revealed an area of decreased metabolism of glucose in the left temporal region (Figure 2) - on the same side as the trauma.

**Diagnostic tools useful for identifying athletes with concussion.** A checklist of symptoms can be assessed by several tools, such as the Post-Concussion Symptom Scale (PCSS) or Graded Symptom Checklist (GSC). These tools are not substitute for more thorough medical, neurologic, or neuropsychological evaluations and cannot be used to exclude the diagnosis of concussion. GSC and PCSS have a sensitivity of 64%-89%, and a specificity of 91%-100% for identifying concussion in athletes.^9,38-41^

There is another instrument called the Standardized Assessment of Concussion (SAC) designed for 6-minute administration which assesses 4 neurocognitive domains - orientation, immediate memory, concentration, and delayed recall. It is for use by non-physicians at the sidelines of athletic events. The SAC can often identify the presence of concussion in the early stages of post-injury (sensitivity of 80%-94%, and specificity of 76%-91%).^9,21,42-45^

Although neuropsychological tests may be administered by non-neuropsychologists, they require a neuropsychologist for accurate interpretation of the results obtained. It is likely that neuropsychological testing of memory performance, reaction time, and speed of cognitive processing - administered by paper-and-pencil or computerized methods - is useful in identifying the presence of concussion (sensitivity 71%-88% in athletes with concussion).^9,46-48^

Other tools include the Balance Error Scoring System (BESS) that assesses postural stability and can be completed in about 5 minutes plus The Sensory Organization Test (SOT) that measures a subject's ability to maintain balance while systematically altering orientation information available to the somatosensory or visual inputs (or both).^9,46^

**Prediction of early post-concussion impairments.** Lower SAC scores,^9,46^ neuropsychological testing score reductions,^38^ and deficits on BESS^9^ and SOT^49^ are likely to be associated with more severe or prolonged early post-concussive cognitive impairments.

**Poor prognosis or diagnosis of catastrophic outcomes.** Prior history of headaches is a possible risk factor for persistent neurocognitive problems.^50^

Possible risk factors for more prolonged return to play include having symptoms of dizziness,^51^ playing as quarterback in football,^52^ and wearing a half-face shield in hockey^53^ (compared to wearing full-face shields). Playing on artificial turf in football is possibly a risk factor for more severe concussions.^20^

Early posttraumatic headache,^38,54^ fatigue/fogginess,^54^ early amnesia, alteration in mental status, or disorientation^39,54^ are probable risk factors for persistent neurocognitive problems or prolonged return to play. Likewise, it is probable that younger age/level of play^24^ is a risk factor for prolonged recovery.

**Increased risk of concussion.** A history of concussion is a highly probable risk factor for recurrent concussion^20,28,32^ - increased risk for repeat concussion in the first 10 days.^31^

Longer length of participation^55^ and quarterback position played in football^52,55^ are additional probable risk factors for recurrent concussion.

**Predictors of chronic neurobehavioral impairment.** Prior concussion exposure is highly likely to be a risk factor for chronic neurobehavioral impairment and there appears to be a relationship with increasing exposure. This holds true for professional sports such as football, soccer, boxing, and horse racing.^56-60^ The data are insufficient to determine whether there is a relationship between chronic cognitive impairment and heading in professional soccer.^61,62^

No conclusions can be drawn with regard to amateur athletes.^55,57^

APOE e4 genotype seems to be associated with chronic cognitive impairment after concussion exposure,^58,63^ and preexisting learning disability may be a risk factor of chronic neurobehavioral impairment.^55^

Sex and age are not established as risk factors for chronic post-concussive impairments owing to a lack of data.^5^

**Management.** The first step after a concussion in sports is to immediately remove the player from play. The player must then be evaluated by a health-care professional.^6-8^ It is recommended that the athlete undergoes a period of cognitive and physical rest until they become asymptomatic.

A gradual stepwise return to competition should be attempted only after the athlete is asymptomatic and no longer receiving medications to treat or modify the symptoms of concussion.^8^ Table 1 shows medications used in the treatment of concussive symptoms.^64-66^

**Table 1:** Medications used to treat concussive symptoms.

Medication	Use
Analgesics, nonsteroidal anti-inflammatories, antidepressants, anticonvulsants, beta-blockers and triptans	For headache
Vestibular suppressants and benzodiazepines	For dizziness
Neurostimulants	For fatigue
Antiemetics	For nausea
Antidepressants	For depression
Anxiolytics	For anxiety
Neurostimulants, selective serotonin reuptake inhibitors, and anticholinesterase inhibitors	For improving neurocognitive performance following TBI

When the athlete is asymptomatic at rest and on exertion they can return to full activity.^6-8^

If an athlete does not show improvement after cognitive and physical rest for a period of time, a low-level - subsymptom threshold rehabilitation and/or exercise programme - may be of benefit in improving post-concussion syndrome (PCS).^67,68^

## CONCLUSION

Acute TBI in sport is an important public health concern in our society. Concussions are recurrently unrecognized and consequently underreported. The mismanagement of a concussion may result in a persistent post-concussion syndrome and/or second-impact syndrome, making recognition and proper medical supervision of concussion vitally important.
